# The New Face of a Well-Known Antibiotic: A Review of the Anticancer Activity of Enoxacin and Its Derivatives

**DOI:** 10.3390/cancers14133056

**Published:** 2022-06-22

**Authors:** Karolina Jałbrzykowska, Alicja Chrzanowska, Piotr Roszkowski, Marta Struga

**Affiliations:** 1 Chair and Department of Biochemistry, Medical University of Warsaw, Banacha 1, 02-097 Warszawa, Poland; kjalbrzykowska@wum.edu.pl; 2 Faculty of Chemistry, University of Warsaw, Pasteura 1, 02-093 Warsaw, Poland; roszkowski@chem.uw.edu.pl

**Keywords:** enoxacin, derivatives, miRNA, prooxidative, anticancer

## Abstract

**Simple Summary:**

Enoxacin is a second-generation quinolone with promising anticancer activity. In contrast to other members of the quinolone group, it exhibits an extraordinary cytotoxic mechanism of action. Enoxacin enhances RNA interference and promotes microRNA processing, as well as the production of free radicals. Interestingly, apart from its proapoptotic, cell cycle arresting and cytostatic effects, enoxacin manifests a limitation of cancer invasiveness. The underlying mechanisms are the competitive inhibition of vacuolar H^+^-ATPase subunits and c-Jun N-terminal kinase signaling pathway suppression. The newly synthesized enoxacin derivatives have shown a magnified cytotoxic effect with an emphasis on prooxidative, proapoptotic and microRNA interference actions. The mentioned mechanisms seem to contribute to a safer, more selective and more effective anticancer therapy.

**Abstract:**

Enoxacin as a second-generation synthetic quinolone is known for its antibacterial action; however, in recent years there have been studies focusing on its anticancer potential. Interestingly, it turns out that compared to other fluoroquinolones, enoxacin exhibits uncommon cytotoxic properties. Besides its influence on apoptosis, the cell cycle and cell growth, it exhibits a regulatory action on microRNA biogenesis. It was revealed that the molecular targets of the enoxacin-mediated inhibition of osteoclastogenesis are vacuolar H^+^-ATPase subunits and the c-Jun N-terminal kinase signaling pathway, causing a decrease in cell invasiveness. Interestingly, the prooxidative nature of the subjected fluoroquinolone enhanced the cytotoxic effect. Crucial for the anticancer activity were the carboxyl group at the third carbon atom, fluorine at the seventh carbon atom and nitrogen at the eighth position of naphyridine. Modifications of the parent drug improved the induction of oxidative stress, cell cycle arrest and the dysregulation of microRNA. The inhibition of V-ATPase–microfilament binding was also observed. Enoxacin strongly affected various cancer but not normal cells, excluding keratinocytes, which suffered from phototoxicity. It seems to be an underestimated anticancer drug with pleiotropic action. Furthermore, its usage as a safe antibiotic with well-known pharmacokinetics and selectivity will enhance the development of anticancer treatment strategies. This review covers articles published within the years 2000–2021, with a strong focus on the recent years (2016–2021). However, some canonical papers published in twentieth century are also mentioned.

## 1. Introduction

Fluoroquinolones are synthetic antibacterial agents, representing the first set of anti-infective agents that were not modeled knowingly after any natural antibiotics [1,2]. Over the years they were modified in order to improve and extend the antimicrobial activity. The first generation of quinolones was banned due to their weak pharmacokinetic properties, narrow range of antibacterial activity and rapidly developing resistance to bacteria. Subsequent changes in the quinolone structure opened the second generation of these drugs, which includes ciprofloxacin, enoxacin and ofloxacin.

**Figure 1:**
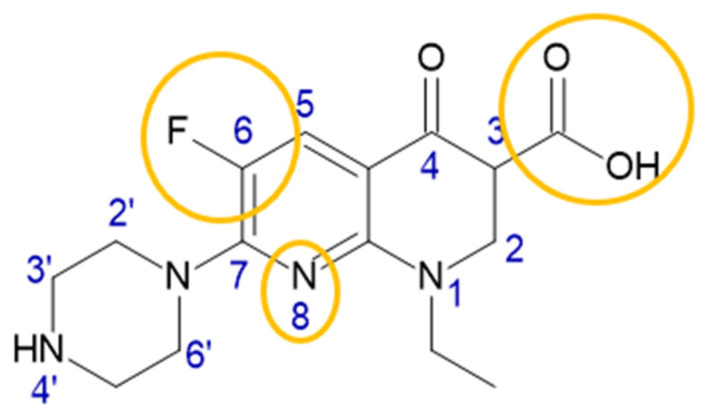
Structure of enoxacin. The figure shows the fragments of the structure responsible for the regulation of miRNA expression.

Enoxacin (ENX) (Figure 1) is an oral broad-spectrum fluoroquinolone bacterial agent, exhibiting high antibacterial activity against a broad spectrum of Gram-negative bacteria and moderate activity against Gram-positive bacteria [3,4,5]. It has been used for many years in clinical practice under the brand name Penetrex for the treatment of genitourinary tract infections [6]. ENX is a 1,8-naphthyridine derivative of 1,4-dihydro-1,8-naphthyridine with an ethyl group at the 1 position, a carboxy group at the 3-position, an oxo-substituent at the 4-position, a fluorine-substituent at the 5-position and a piperazin-1-yl group at the 7 position. Enoxacin inhibits bacterial DNA gyrase and topoisomerase IV [7,8]. Reports concerning its ability to interfere with human topoisomerase are contrary [1,8,9,10] and it seems that this interaction is concentration-dependent [8].

Fluoroquinolones are a group of substances that have been thoroughly tested in terms of their anticancer activity. Only in recent years have a number of reviews describing the cytotoxic properties of fluoroquinolones been published [11,12,13,14].

Like most fluoroquinolones, enoxacin has been tested for its anticancer activity. However, distinct activities of enoxacin, such as anti-ALS, antidepressant, antiaging and antiviral activities, are reported in literature [9]. In this review, we focus on the anticancer potential as well as the mechanisms of anticancer activity for enoxacin and its derivatives.

## 2. Enoxacin as a Small-Molecule Enhancer of microRNA (SMER)

### 2.1. The miRNA Biogenesis

**Figure 2:**
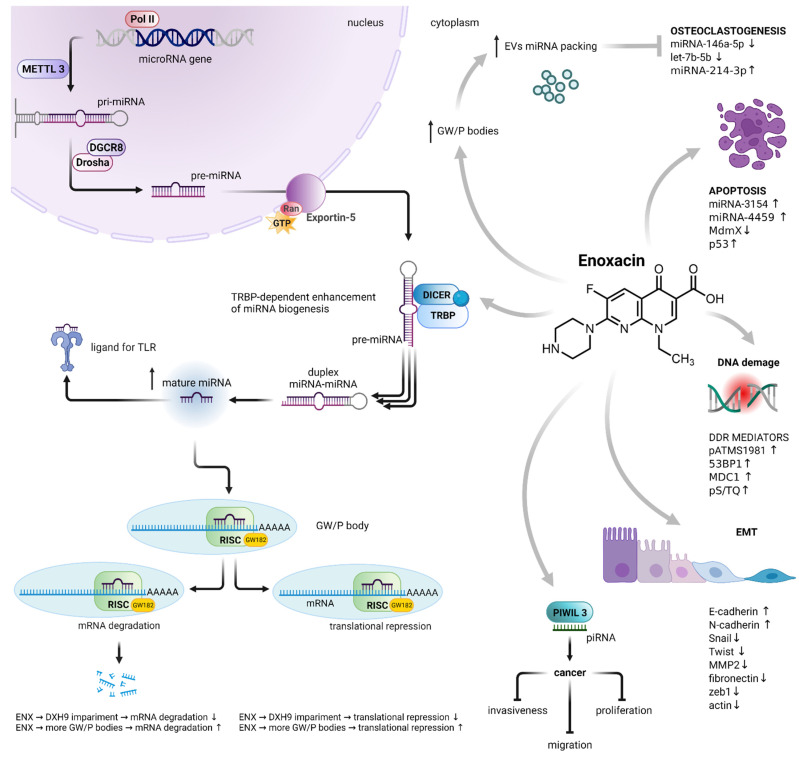
Enoxacin-induced dysregulation of miRNA biogenesis and its consequences. Enoxacin enhanced the activity of the RISC loading protein TRBP, resulting in an increase in the number of mature miRNAs. It also impairs the activity of DHX9 helicase, a member of the RISC, leading to the impairment of mRNA translational repression and degradation. On the other hand, it increases the number of GW/P bodies, the sites of RNA-mediated silencing, as well as the localization of miRNA packaging into EVs. The enoxacin-dysregulated biogenesis of miRNA is involved in osteoclastogenesis, apoptosis, DNA damage response, epithelial–mesenchymal transition, cancer cell proliferation, migration and invasiveness. Abbreviations: polymerase II (Pol II); methyltransferase-like 3 (METTL3); microprocessor complex subunit DGCR8 (DGCR8); type III RNase Drosha (Drosha); GTP-binding nuclear protein Ran (Ran); endoribonuclease DICER (DICER); TAR RNA-binding protein 2 (TRBP); Toll-like receptor (TLR); RNA-induced silencing complex (RISC); GW processing body (GW/P-body); Piwi-interacting RNA (piRNA); epithelial–mesenchymal transition (EMT); matrix metalloproteinase-2 (MMP2); DNA damage response (DDR); extracellular vesicles (EVs). MiRNA biogenesis adapted from [16]. Created with BioRender.com.

The miRNA biogenesis begins with transcribing a gene into a large primary transcript (pri-miRNA). The transcription is typically mediated by RNA polymerase II [15]. The pri-miRNAs are then cleaved by the microprocessor complex, composed of the RNA-binding protein DGCR8 and type III RNase Drosha, into a stem-loop structure called the precursor miRNA (pre-miRNA) [16]. Following transportation by the Ran/GTP/Exportin5 complex from the nucleus to the cytoplasm, the pre-miRNAs are processed by another RNase III enzyme DICER into an miRNA/miRNA duplex. After the duplex is unwound, the mature miRNA is incorporated into a protein complex termed the RNA-induced silencing complex (RISC). The miRNA-loaded RISC mediates gene silencing via mRNA cleavage and degradation or translational repression, depending on the complementarity between the miRNA and the targeted mRNA transcript [17]. An important role in translational repression and mRNA degradation is played by GW182 protein. Its N-terminal domain is rich in glycine (G) and tryptophan (W) amino acids, while the C-terminus contains a silencing domain. GW182 directs proteins involved in deadenylation, decapping and exonucleolytic degradation of the target mRNA [18]. GW182 binds directly to AGO2, another member of the RISC [19] GW182, AGO2 and rest of the RISC can be found within GW processing bodies (GW/P-bodies) [20,21]. Interestingly, miRNAs may function as ligands directly binding the Toll-like receptor (TLR), triggering downstream signaling pathways [22] (Figure 2). 

It has been clear for almost twenty years that miRNA expression is dysregulated in human malignancies. The underlying mechanisms include chromosomal abnormalities [23,24,25,26,27,28], transcriptional control changes [29,30,31], epigenic changes and defects in the miRNA biogenesis machinery [32,33,34]. 

### 2.2. The Effect on Cancer Cells

#### 2.2.1. TRBP-Dependent Cytotoxicity

Small molecules can influence miRNA biogenesis. Enoxacin was reported as the first and unique small-molecule enhancer of microRNA (SMER) maturation [35]. 

Jin et al. found that the increase in miRNAs was associated with high levels of their own precursors, thereby suggesting that enoxacin could promote the DICER processing activity without influencing the miRNA precursor expression [35]. The same authors established that the enoxacin activity was dependent on the TAR RNA-binding protein 2 (TRBP) and likely involved in the improvement of the TRBP-pre-miRNA affinity [35].

The role of the TRBP in enoxacin-mediated cytotoxicity was confirmed in the three colorectal cancer cell lines (Co115, RKO and HCT-116) and their mutants with impaired TRBP expression. TRBP-impaired cells were more resistant to enoxacin, resulting in a 2-fold increase in the effective concentration (EC50). Mutant cells also did not undergo cell cycle arrest. Additionally, enoxacin induced a two-fold expression increase in 24 miRNAs in RKO and HCT-116 cells and from 1.5-fold up two five-fold increases in the expression of those miRNAs in RKO and HCT-116 xenograft mouse model. Enoxacin treatment also significantly decreased the number of lung and liver metastases in the HCT-116 xenograft [36]. Interestingly, ENX increased the TRBP-dependent miRNA expression in Ewing’s sarcoma family tumor (ESFT) cell lines such as A673, TC252 and STA-ET-8.2, but not the expression of TRBP itself. It caused a 50% reduction in sphere formation; increased the expression of a panel of TRBP/DICER-dependent miRNAs; and decreased the expression of Oct-4, Nanog and Sox-2 proteins in primary ESFT spheres. This effect was similar to the introduction of exogenous TRBP. Thus, enoxacin can cause a decrease in the self-renewal of ESFT cancer stem-like cells (CSC) [37]. However, this fluoroquinolone also significantly decreased TRBP and DICER protein expression levels in the prostate cancer cell lines DU145, LNCaP, VCaP, PC-3, 22Rv1 and Co115 via the induction of apoptosis. At the same time, it dysregulated the expression of a wide range of miRNAs involved in the development and progression of prostate cancer, e.g., miRNA-29b, which regulates the expression of the proteins E-cadherin, N-cadherin, Snail, Twist and matrix metalloproteinase-2 (MMP2) involved in the metastatic process [38]. The effect of the increased expression of tumor-suppressing miRNA was also notable in thyroid cancer cells lines (Cal62, TPC1, SW1736). This resulted in lower cell proliferation and cell invasiveness in vitro. The decreased expression of epithelial–mesenchymal transition (EMT) markers such as fibronectin, n-cadherin, zeb1, twist and actin was also observed. The upregulation of tumor suppressor miRNA, as well as the suppression of EMT markers, was also shown in the orthotopic mouse model of human thyroid cancer. The results were consistent with those using miRNA-restoring DICER1 silencing (miRNA-30a and miRNA-100). This suggests that enoxacin promotes the restoration of DICER1 activity in DICER1-impaired cells, e.g., human thyroid cancer cells [39].

The role of enoxacin as an enhancer of DNA damage response (DDR) signaling was confirmed in cervical cancer HeLa cells. This antibiotic augmented the DDR mediator factors pATMS1981, 53BP1, MDC1 and pS/TQ without affecting their expression, whereas the activity of γH2AX levels was not affected. Based on the knowledge of the activation of TRBP by enoxacin, it is not surprising that TRBP silencing diminished ENX’s stimulation of DDR. The knockdown of PACT or GW182 proteins (TNRC6A, B and C), which are effectors for miRNA-guided gene silencing, did not affect ENX-mediated DDR stimulation. These results seem to strengthen and confirm enoxacin’s specificity towards TRBP [40].

#### 2.2.2. PIWIL-3-Dependent Cytotoxicity

Regarding the molecular target recognized by enoxacin, an additional protein has been proposed that merits mentioning. In 2017, Abell et al. determined that the Piwi-like protein 3 (PIWIL3) is a potential enoxacin target [41]. PIWIL3 belongs to the PIWI argonaut proteins involved in the maturation of the PIWI-interacting RNAs (piRNAs), small non-coding RNAs that differ from miRNAs [42]. Although mostly present in normal testis tissue, PIWIL3 has been reported to be aberrantly expressed in a variety of cancers, playing important roles in tumorigenesis [43,44]. An increase in miRNA-21 and miRNA-145 expression was observed in breast cancer MCF7 cells. Similar results were obtained in cells with small interfering RNA-mediated knockout of the PIWIL3. The staining with alkenox, a synthetic enoxacin analog, showed that PIWIL3 might be a mechanistic target of enoxacin [41]. Since PIWIL3 is more abundant in cancer cells, it might in part explain the specificity of ENX towards cancer cells [41,44].

#### 2.2.3. Other Consequences of Enoxacin-Mediated miRNA Dysregulation

Additionally, ENX affected RNA helicase DHX9, a member of the RISC. The expression of DHX9 was much higher in the small-cell lung cancer cell line H446 than in the non-small-cell lung carcinoma cell lines A549 and PC9. Enoxacin inhibited the proliferation of A549 in a dose-dependent manner. Similarly, it decreased the expression of DHX9 in A549 cells. However, silencing DHX9 impaired the cytotoxic effect of the drug [45]. 

A strong inhibitory effect of enoxacin on the proliferation of human melanoma A375, Mel-Juso and Mel-Ho cell lines was observed. It dysregulated a set of 55 miRNAs in A375 cells (26 upregulated, 29 downregulated). Two upregulated miRNAs, miRNA-3154 and miRNA-4459, control the p53-Mdm2-MdmX network [46]. Interestingly, in many melanomas the overexpression of MdmX, a p53 negative regulator, was observed [47]. Enoxacin increased p53’s activity without affecting its expression. At the same time, the expression of MdmX decreased in a dose-dependent manner. It alternates MdmX splicing by promoting exon 6 skipping. This process was observed in different cancer cell lines (A375, A2780 and MCF7) [48]. 

A different ENX antiproliferative mechanism without affecting apoptosis has been noted in 4T1 murine breast cancer cells. An increased level of GW/processing bodies, which are considered the surrogate markers for both the microRNA-mediated repression of translation and the extracellular vesicle (EV) packaging sites, was observed. MiRNA expression levels in the 4T1 cells’ cytosol and their EV were compared. Enoxacin significantly increased only miRNA-214-3p and slightly increased miRNA-146a-5p, miRNA-290, miRNA-689 and let-7b-5p cytosolic levels. In contrast, significant decreases in let-7b-5p, miRNA-146a-5p and miRNA-689 expression and an enormous 22-fold increase in miRNA-214-3p were observed. All mentioned miRNAs are involved in the regulation of bone remodeling and osteoclastogenesis. Additionally, EVs from enoxacin-treated 4T1 cells enhanced the proliferation of murine macrophage cells [49]. The effective concentration range affecting miRNA biogenesis (see Table 1) was from 50 to 124 µM; however, an achievable serum concentration for a standard clinical dose 400 mg two times a day was ca. 10 µM. Reduction of effective concentration could be obtained by structural modification of enoxacin. Significant decrease of IC50 was observed after structure modification of other fluoroquinolones, i.e., by fatty acid conjugation [50,51]. Moreover, it should be considered that tumor vessels are often more permeable compared to normal vessels, which could increase the ENX delivery and its intratumor concentration [52].

Although it has been clear that enoxacin did not affect all existing miRNAs (e.g., 36 out of 22,000 tested in normal HEK293 cells [35] and 122 out 731 tested in cancer RKO cells [36]). It is worth mentioning that this may be partially explained by the existence of the alternative miRNA biogenesis pathways, which do not contain molecular targets of enoxacin [53,54,55]. Interestingly, the dysregulated miRNAs play an important role in cancer-related processes, e.g., miR-17* decreased the activity of mitochondrial antioxidative enzymes in PC3 cells [56]; miR-34a downregulated an oxidative-stress-induced silent information regulator 1 (SIRT1), a negative regulator of p53 protein, in HCT116 cells [57]; miR-30a-5p suppressed the epithelial–mesenchymal transition in SW480 cells by targeting integrin β3 (ITGB3) [58]; and miR-212 was observed to inhibit the viability and invasion of HCT116 and SW620 cells via inhibition of the phosphoinositide-3-kinase regulatory subunit 3 (PIK3R3) expression [59]. An investigation of the role of the dysregulated miRNAs would definitely help to better understand the mechanism of enoxacin-mediated anticancer activity. However, the detailed molecular targets of particular tumor-suppressing or oncogenic miRNAs are beyond the scope of this review and have been described elsewhere [60,61,62,63,64]. 

### 2.3. The Effects on Non-Cancer Cells

It is worth mentioning that enoxacin affects not only cancer cells; it also increased the levels of miRNA related to the disease in the dominant negative TGF-β receptor (dnTGFβRII) CD8 cells from an autoimmune cholangitis mouse model. Despite the fact that enoxacin did not change the amount of CD8 T cells, it significantly decreased their proliferative response. Enoxacin also significantly decreased the level of interferon γ in mouse serum [65]. There is some research concerning enoxacin’s impact on the neuronal system. Rats treated with 10 or 25 mg/kg enoxacin for 1 week were found to have elevated levels of miRNA related to the neuronal cell biology in their frontal cortex (Table 2). Those miRNAs include let-7, miRNA-124, miRNA-125 and miRNA-132. They are involved in the processes of neurogenesis (let-7, miR-124) and neuronal differentiation in human (miRNA-125) and mouse (miRNA-124) brains, the regulation of the dendritic spine length in mammalian neurons (miR-125NA), as well as neurite outgrowth (miRNA-132). The enoxacin-treated rats were also less likely to exhibit learned helplessness when they faced an inescapable shock [66].

Enoxacin is also capable of affecting artificial miRNAs (amiRNAs), especially by enhancing the amiRNA-mediated reversible inhibition of the CRISPR-Cas9 system in both in vitro and in vivo studies. Interestingly, amiRNA alone did not show an inhibitory effect towards the CRISPR-Cas9 system, indicating the crucial role of enoxacin. In contrast, some of the naturally occurring miRNAs were able to inhibit CRISPR-Cas9 activity by binding to single-guide RNA (sgRNA), a part of the sgRNA/Cas9 complex, in the absence of enoxacin. Surprisingly, the presence of enoxacin in concentrations up to 50 µM did not affect the natural miRNAs’ influence on the CRISPR system. The proposed explanation of these differences in the impacts of enoxacin on the inhibition of natural and artificial miRNAs is based on the low binding capacity of amiRNAs towards RISC and the high binding capacity towards the RISC of natural miRNAs. Another factor could be the difference in the amounts of amiRNAs vs. natural miRNAs. The amiRNAs are believed to outnumber natural miRNAs. Taking the above into account, the amiRNA/enoxacin system turned out to be specific and reversible, making it a convenient tool for CRISPR-Cas9 regulation [67].

In 2008, Shan et al. investigated the ability of microRNA processing to enhance some fluoroquinolones, including enoxacin and its three derivatives. They identified the structural elements of enoxacin responsible for its activity, such as a carboxyl group at the 3rd carbon atom, fluorine at the 7th carbon atom, as well as nitrogen at the 8th position of naphyridine. [35].

**Table 1:** The miRNA-regulating activity levels in in vitro studies depending on the different enoxacin concentrations.

miRNA	Conc. [µM]	Effect	Expression Change	Cell Line	Ref.
		[↑/↓]	Change-Fold		
Cancer cells					
let-7b-5p, miR-146a-5p, miR-689	50	↓	0.5–1	4T1 (miRNA from EV),	[49]
miR-100	124	↓	0.5–1	primary ESFT spheres	[37]
miR-141, miR-191	124	↓	1.5–2	DU145, LNcap,	[38]
miR-21-5p, miR-30a-3p, miR-30a-5p, miR-100-5p, miR-204-5p, miR-221-3p	124	↑	<1.5	Cal62, STA-ET-8.2, TPC1	[39]
Let-7f, miR-26a,	124	↑	<1.5	A673, SW1736	[37]
miR-21	100	↑	1.5–2	MCF7	[41]
miR-16, miR-18a*, miR-21, miR-26a, miR-29b, miR-29c, miR-31, miR-193a,	124	↑	1.5–2	HCT-116	[36]
let-7f, miR-26a, miR-99a, miR-100, miR-143, miR-145,	124	↑	1.5–2	A673, STA-ET-8.2, TC252, primary ESFT spheres	[37]
miR-21-5p, miR-30a-3p, miR-100-5p, miR-146b-5p, miR-221-3p,	124	↑	1.5–2	Cal62, SW1736, TPC1	[39]
miR-17 *, miR29b, miR-132, miR-146a, miR-191 miR-449a,	124	↑	1.5–2	DU145 LNcap,	[38]
miR-214-3p	50	↑	2–2.5	4T1 (cytosolic miRNA),	[49]
miR-145	100	↑	2–2.5	MCF7	[41]
miR-7, miR-16, miR-18a*, miR-29c, miR-101, miR-128, miR-181a, miR-212	124	↑	2–2.5	HCT-116, RKO	[36]
miR-100-5p, miR-146b-5p	124	↑	2–2.5	SW1736, TPC1	[39]
miR-34a, miR-449a	124	↑	2–2.5	DU145, LNcap	[38]
let-7f, miR-99a, miR-100, miR-145	124	↑	2–2.5	A673, STA-ET-8.2, TC252, primary ESFT spheres	[37]
miR-7, miR-26a, miR-29b, miR-30a, miR-101, miR-122, miR-125a, miR-125b, miR-126, miR-128, miR-143, miR-181b, miR-205	124	↑	2.5–3	HCT-116, RKO	[36]
miR-100, miR-145	124	↑	2.5–3	A673, TC252	[37]
miR-29b	124	↑	2.5–3	LNcap	[38]
let-7a, let-7b, miR-30a, miR-31, miR-126, miR-181b, miR-193a, miR-193b,	124	↑	3–3.5	HCT-116, RKO	[36]
let-7f, miR-143, miR-181a,	124	↑	3–3.5	A673, STA-ET-8.2, primary ESFT spheres	[37]
miR-181a, miR-193b	124	↑	3.5–4	HCT-116	[36]
let-7b, miR-143, miR-205	124	↑	4–4.5	HCT-116, RKO	[36]
miR-143	124	↑	4–4.5	TC252	[37]
miR-125a	124	↑	ca. 5	HCT-116	[36]
miR-214-3p	50	↑	ca. 22	4T1 (miRNA from EV)	[49]
Non-cancer cells					
miR-128-1	60	↓	0.5–1	dnTGFβRII T cells	[65]
let-7i, miR-128	50	↓	1.5–2	HEK293	[35]
let-7b, miR-23a, miR-30e, miR-96, miR-99a, miR-125a, miR-146, miR-190, miR-199a*,	50	↑	1.5–2	HEK293	[35]
miR-124a, miR-139, miR-152, miR-199b	50	↑	2–2.5	HEK293	[35]
miR-29b-1, miR-145a-5p, miR-326-3p	60	↑	2–2.5	dnTGFβRII T cells	[65]
miR-181a	60	↑	2.5–3	dnTGFβRII T cells	[65]
miR-346-5	60	↑	3–3.5	dnTGFβRII T cells	[65]

Dominant negative TGF-β receptor (dnTGFβRII). Please note that “*” is not a footnote indicator, but an essential part of the name of the specific miRNA. More information regarding the miRNAs’ nomenclature can be found in miRBase [68].

**Table 2:** The miRNA regulating activity levels in in vivo studies depending on the different enoxacin concentrations.

miRNA	Dose	Effect	Expression Change:	Tissue	Ref.
		[↑/↓]	Change-Fold		
miR-124	10 mg/kg 25 mg/kg	↑	ca. 4. ca. 6	rat frontal cortex	[66]
let-7a, miR-125a-5p	10 mg/kg 25 mg/kg	↑	ca. 11. ca. 20		
miR-132	10 mg/kg 25 mg/kg	↑	ca. 19 (for both doses)		
miR-30a-5p, miR-146b-5	15 mg/kg	↑	1.5–2	human orthotopic thyroid tumor from Cal62-luc mouse	[39]
mIR-100-5p, miR-30-3p, miR-204-5	15 mg/kg	↑	2–2.5	human orthotopic thyroid tumor from Cal62-luc mouse	[39]
miR-16, miR-18a*, miR-21, miR-26a, miR-29b, miR-29c, miR-31, miR-101, miR-193a	10 mg/kg	↑	1.5–2	tumor from HCT-116 mouse xenograft	[36]
miR-16, miR-29c, miR-31, miR-101, miR-181a	10 mg/kg	↑	1.5–2	tumor from RKO mouse xenograft	[36]
miR-128, miR-212	10 mg/kg	↑	2–2.5	tumor from HCT-116 mouse xenograft	[36]
miR-18a*, miR-21, miR-26a, miR-29b, miR-30a, miR-128	10 mg/kg	↑	2–2.5	tumor from RKO mouse xenograft	[36]
let-7b, miR-7, miR-143, miR-181b, miR-125b	10 mg/kg	↑	2.5–3	tumor from HCT-116 mouse xenograft	[36]
let-7a, miR-7, miR-122, miR-125a, miR-125b, miR-126, miR-181b, miR-193a, miR-193b, miR-205, miR-212	10 mg/kg	↑	2.5–3	tumor from RKO mouse xenograft	[36]
let-7a, miR-30a, miR-122, miR-126	10 mg/kg	↑	3–3.5	tumor from HCT-116 mouse xenograft	[36]
miR-143	10 mg/kg	↑	3–3.5	tumor from RKO mouse xenograft	[36]
miR-125a, miR-181a, miR-193b	10 mg/kg	↑	3.5–4	tumor from HCT-116 mouse xenograft	[36]
let-7b	10 mg/kg	↑	4.5–5	tumor from RKO mouse xenograft	[36]
miR-205	10 mg/kg	↑	4.5–5	tumor from HCT-116 mouse xenograft	[36]

## 3. Cytotoxic Effects of Enoxacin Mediated by Other Mechanisms 

### 3.1. Effect on Cancer Cells

**Figure 3:**
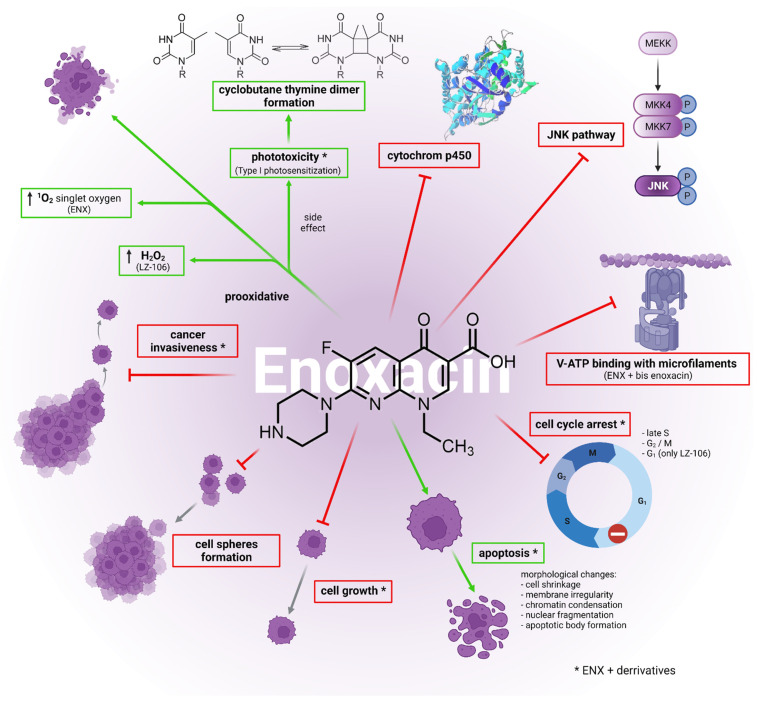
A summary of anticancer mechanisms other than the dysregulation of miRNA biogenesis mediated by enoxacin and its derivatives. Enhanced processes such as prooxidative activity and the induction of apoptosis are marked in green. Inhibition is marked in red. Abbreviations: c-Jun N-terminal kinase (JNK); vacuolar H^+^-ATPase (V-ATPase).

Enoxacin affected cancer cells via various mechanisms (Figure 3). It induced apoptosis in the human non-small-cell lung cancer lines H460 and A549 [69]. Growth inhibition and various morphological changes, including cell shrinkage, membrane irregularity, chromatin condensation, nuclear fragmentation and apoptotic body formation, were observed in H460 cells. Interestingly, growth inhibition was retained even after replacing the drug-free medium [70]. Similar effects, including morphological changes and cell growth inhibition, were observed in the human breast cancer MCF-7 cell line. The effect was dose-dependent but not time-dependent. The flow cytometry analysis (FACS) showed that enoxacin caused cell cycle arrest [71]. An investigation of ENX’s impact on the invasiveness of DU145 cells confirmed its inhibitory nature. A study on HCT-116 and RKO cell lines also proved the induction of cell cycle arrest [36]. ENX was also an inducer of apoptosis in the human non-small-cell lung cancer cell lines H460 and A549, as well as in different prostate cancer cell lines (DU145, LNCaP, VCaP, PC-3, 22Rv1, Co115). It caused G2/M cell cycle arrest in hormone-responsive 22Rv1 and VCaP cell lines, while it caused an increase in the percentage of cells at late S and G2/M transition phases of castration-resistant DU145 and PC-3 cell lines [38]. A significant decrease in MMP and increases in cytochrome c release and elevated caspase-3/9 activities were observed in PC-3 cells by Xu et al. Additionally, they showed cleaved caspase-3, cleaved caspase-9, pro-apoptotic protein Bax and NOXA expression increases, as well as antiapoptotic protein Bcl-2 and MCL-1 expression decreases [72]. Another study confirmed that the anticancer activity significantly decreased the mitotic index and increased the apoptotic index values of both HeLa and C33A human cervical cancer cells. The apoptotic effect was much stronger when enoxacin was incubated with epigallocatechin gallate [73]. 

The subjected fluoroquinolone affected Ewing sarcoma family tumor (ESFT) cells in various ways. It effectively inhibited the growth of ESFT cells derived from primary tumors, but only those that were CD133-positive. Interestingly, the inhibition occurred only in cells cultured in spheres but not in those cultured in a monolayer. It also inhibited the formation of ESFT cell spheres (with the exception of cells derived from a tumor resistant to all conventional treatments), which could be considered as the inhibition of self-renewal. Interestingly, ENX did not affect A673 ESFT cells or primary human pediatric mesenchymal stem cells (hpMSCs) and had little effect on TC252 ESFT. [74]. Consistent results were obtained by testing higher concentrations of enoxacin. A cytotoxic effect was observed in A673, TC252 and STA-ET-8.2 cells. It also inhibited the growth of a tumor originated from an injection of A673 or TC252 cells into mice [37]. Interestingly, enoxacin showed an inhibitory nature towards cytochrome P450. It strongly inhibited the formation of 1,3-dimethyluric acid in freshly isolated rat hepatocytes but did not cause significant changes in rat liver microsomes [75]. The elevated levels of ER stress markers such as ATF6, CHOP, GRP78 and IRE1 were observed in PC-3 cells after enoxacin treatment [72]. It was proven that the drug caused the inhibition of the binding between vacuolar H^+^-ATPase (V-ATPase) and microfilaments. The affected subunits of V-ATPase were B2 subunit [76] and a3 subunit [77]. The proper function of V-ATPase is essential for osteoclast bone resorption [78,79]. Increased expression of V-ATPase was found in a wide range of cancer cell lines, while V-ATPase inhibitors decreased cancer cell invasiveness [80]. However, knockout of the V-ATPase a2 isoform increased the invasiveness of breast cancer in a mouse model [81]. Another mechanism of osteoclastogenesis inhibition by ENX was identified as c-Jun N-terminal kinase (JNK) signaling suppression [82]. Interestingly, overexpression of JNK has been observed in various cancers, and introducing JNK inhibitors resulted in anticancer effects [83]. The above-mentioned mechanisms, such as impairment of the mitochondrial dehydrogenase pathway (MTT assay), cytochrome P450 inhibition, the inhibition of osteoclastogenesis, the inhibition of bone resorption and the inhibition of the interaction between V-ATPase and microfilaments, were observed for clinically relevant concentrations (up to ca. 10 µM) [84]. Surprisingly, despite the clear anticancer potential, there are no clinical trials regarding the anticancer activity of enoxacin according to the information from publicly available databases [85,86,87]. However, there is promise for the therapeutic application of ENX in bladder cancer due to the high urine drug concentrations (ca.1000 µM) substantially exceeding IC50 in a bladder cancer cell line [88]. All details regarding the other cytotoxic mechanisms and concentrations are summarized in Table 3.

Interestingly, enoxacin also played a protective role in irinotecan therapy. It can serve as a strong inhibitor of β-glucuronidase-mediated SN-38-G deconjugation. SN-38 (7-ethyl-10-hydroxy-camptothecin) is a metabolite of Irinotecan. SN-38-G is conjugated in the gastrointestinal tract with glucuronic acid, which prevents side effects of free SN-38, e.g., diarrhea. Enoxacin exhibited the strongest inhibition of deconjugation among the other tested agents [89]. Interestingly, enoxacin did not show significant changes in the human lymphoma WTK-1 cell line, causing no genotoxicity even at a concentration exceeding 3000 μM [90]. 

**Table 3:** Cytotoxic effects of enoxacin depending on the concentration in various cancer cell lines and animal tissues.

Effect	Conc. [μM]	Cell Line/Tissue	Ref.
No cytotoxicity	31	A673	[74]
	124	Wi-38, MRC-5	[36]
	20	HEK 293	[91]
	5–100	Primary BMMs	[82]
	5–100	Raw 264.7	[77]
No genotoxicity	0–3121	WTK-1	[90]
Apoptosis	62	NCI-H460	[70]
	150	H460, A549	[69]
Apoptosis, cell cycle arrest	31–156	MCF-7	[71]
	20–80	HeLa, C33A	[73]
	124	DU145, LNCaP, VCaP, PC-3, 22Rv1, Co115	[38]
	124	HCT-116, RKO	[36]
Cytotoxicity (PI)	78–312	A375	[48]
Cytotoxicity (MTS)	31	TC 252, Patient derived ESFT spheres	[74]
Cytotoxicity (MTT)	124	HCT-116, RKO, HepG2, SNU-1, SNU-638, MDA-MB 231, MCF-7, H23, H1299, A549, MDA-MB-231, HepG2, KG1a, RAJI	[36]
	124	A673, TC252, STA-ET-8.2	[37]
	156	A375, Mel-Juso, Mel-Ho	[48]
	12.5–100	A549	[45]
Decreased invasiveness	124	DU145	[38]
Cytochrome P450 inhibition	10–1000	freshly isolated rat hepatocytes	[75]
Inhibition of osteoclastogenesis	10; 100	Primary MMO	[76]
	10–100	Primary MMO, Raw 264.7	[77]
	5; 10; 50	Primary BMMs	[82]
Inhibition of bone resorption	10; 25; 100	Primary MMO	[76]
	5; 10; 50	Primary BMMs	[82]
Inhibition of the interaction between V-ATPase B-subunit and F-actin	10; 25; 100	rabbit muscle actin and the Vma2p-MBP microfilaments	[76]
Inhibition of the interaction between microfilaments and V-ATPase (a) B2-subunit (b) a3-subunit	50	Primary MMO	[77]
Impairment of JNK signaling	25	Raw 264.7	[82]

Abbreviations: propidium iodide (PI); Ewing sarcoma family tumor (ESFT); mouse marrow osteoclasts (MMO); bone-marrow-derived macrophages (BMMs); maltose-binding protein (MBP); vacuolar H^+^-ATPase (V-ATPase).

### 3.2. Effects on Normal Cells

Enoxacin inhibited cell growth in a panel of cancer cell lines but not in normal cell lines, including normal lymphocytes [36]. Fedorowicz et al. did not observe the cytostatic effect of enoxacin on human embryonic kidney HEK 293 cells [91]. Moreover, ENX was found to inhibit osteoclastogenesis and bone resorption in primary mouse marrow cells and Raw 264.7 cells [76,77]. 

## 4. Phototoxicity

### 4.1. Mechanism and Effect on Cancer Cells

**Figure 4:**
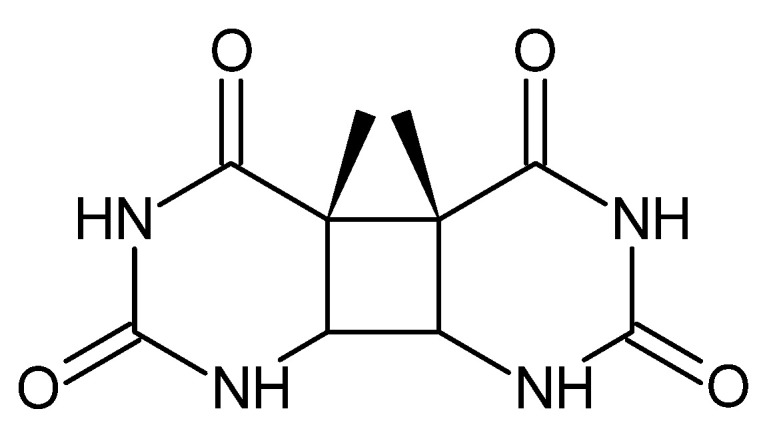
Cyclobutane thymine dimers (thymine dimers).

Enoxacin was found to enhance the cytotoxicity in vitro when cells were irradiated with UVA light in comparison to cells treated with UVA alone (Table 4). The fluoroquinolone induces significantly more cyclobutane thymine dimers (Figure 4) and increases diol formation while only slightly increasing 8-Oxo-7,8-dihydro-2′-deoxyguanosine (8-oxodGuo) formation. However, lomefloxacin and norfloxacin induced much more thymidine dimers than enoxacin. These results suggest that enoxacin induces type-I photosensitization, which is the result of a direct reaction of the excited photosensitizers with DNA via either electron or hydrogen removal. On the other hand, the type-II photosensitization mechanism involves energy transfer from triplet-excited quinolone to molecular oxygen. Both mechanisms lead to the oxidation of guanine, which results in the formation of 8-oxodGuo [92]. 

Yamamoto et al. studied the phototoxicity level against HeLa cells using eight different fluoroquinolones, and enoxacin exhibited almost the highest photocytotoxicity index. Only ofloxacin performed at a higher level (a 2.3-fold higher index than enoxacin), while gatifloxacin showed nearly 42 times lower photocytotoxicity than enoxacin. Sparfloxacin’s index was almost equal to the enoxacin’s index [93]. Interestingly, enoxacin incubated together with δ-aminolevulinic acid (ALA) induced not only phototoxicity but also increased ALA-induced protoporphyrin accumulation in human epithelial cervical cancer HeLa and epidermoid carcinoma A431 cells [94]. A further investigation of the phototoxic mechanism showed that the cytotoxic effect of the UVA irradiation of enoxacin-treated human pancreatic cancer AsPC1 cells was diminished by incubation with histidine and NaN_3_ (singlet oxygen scavengers), partially via incubation with mannitol (a hydroxyl radical scavenger). The incubation with superoxide dismutase (a superoxide anion scavenger) did not influence the enoxacin-UVA-treatment-induced apoptosis. This result suggests the important role of singlet oxygen in enoxacin-induced photocytotoxicity [95]. Similar results were obtained for the enoxacin–UVA treatment of human promyelocytic leukemia HL-60. This fluoroquinolone enhanced the cytotoxic effect of the UVA irradiation. An investigation of different reactive oxygen species (ROS) scavengers showed that mannitol (a hydroxy radical scavenger) and superoxide dismutase (a superoxide anion scavenger) did not affect the apoptotic effects of the enoxacin–UVA treatment. However, NaN_3_ (a singlet oxygen scavenger) significantly decreased the percentage of apoptotic cells. This supports the idea of the role of singlet oxygen in enoxacin-induced photocytotoxicity [96].

**Table 4:** Phototoxic effects of enoxacin depending on the UVA irradiation dose in in vitro studies.

Effect	UVA Irradiation Dose [J/cm^2^]	Conc. [μM]	Cell Line	Ref.
Photosynthetization	3; 6	62	The THP-1 cells from tumoral monocytes	[92]
Phototoxicity	4.3	4.5	HeLa	[93]
Photohemolysis	20	3.1; 31; 312	Sheep red blood cells	[93]
DNA strand breaking	1.6	3.1; 31; 312	pUC18 plasmid was	[93]
apoptosis	4	100	HaCaT	[97]
Increased protoporphyrin accumulation and photosensitivity	0.54 0.54	156–312 (with 1 mM ALA) 312 (with 1 mM ALA)	HeLa A431	[94]
Apoptosis	Approx. 0.84 ^	100	AsPC1	[95]
Apoptosis	0.84	200	HL-60	[96]

^ The precise dose was not mentioned but irradiation parameters were the same as described in [80]; δ-aminolevulinic acid (ALA).

### 4.2. Mechanism and Effect on Normal Cells

Enoxacin caused photosensitization in human keratinocyte HaCaT cells, inducing apoptosis, which was confirmed via fluorescence-activated cell sorting (FACS) analysis and elevated caspase-3 levels [97]. Also shown was a dose-dependent photohemolytic effect in sheep red blood cells. This was the second-strongest effect, lower only than the ciprofloxacin-induced effect. Enoxacin also turned out to be the strongest plasmid pUC18 DNA strand breaker [93].

## 5. Enoxacin Derivatives and Their Anticancer Activity

**Figure 5:**
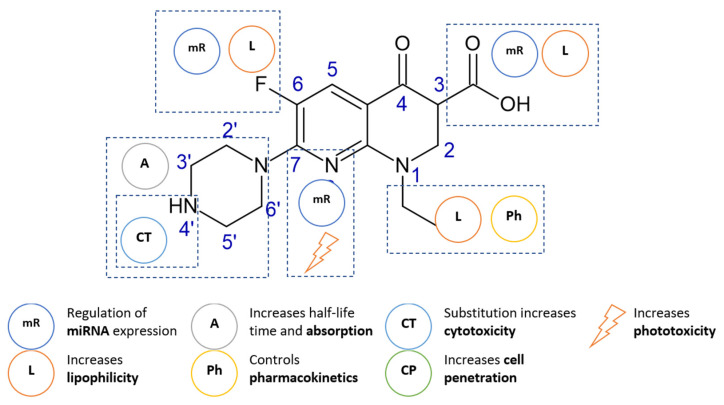
The essential structure requirements of enoxacin as an anticancer agent.

Over the years, many attempts have been made to modify the chemical structure of the fluoroquinolones in order to increase their antimicrobial activity or alter their profile of action. Chemical modifications of enoxacin resulting in an increase in the cytotoxic potential were carried out at the carboxyl group (position 3), the nitrogen atom (position 1) or at the nitrogen atom of the piperazine substituent (position 7). There are many enoxacin diagrams describing the importance of individual structural elements in the literature [98,99,100]. The information relevant to the cytotoxic activity is summarized in the diagram below (Figure 5).

**Figure 6:**
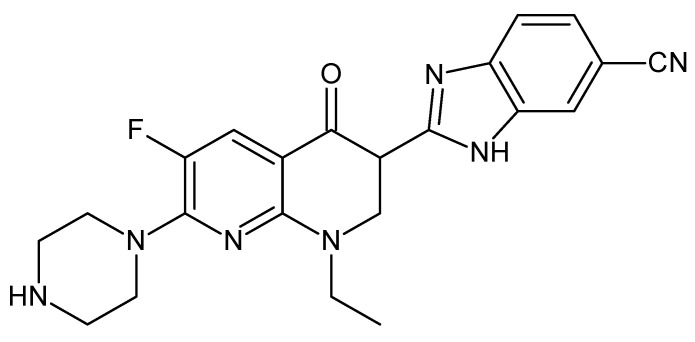
Chemical structure of LZ-106.

Dr. Zhiyu Li synthesized the enoxacin derivative by replacing the carboxyl group with a 2,3-dihydro-1H-benzimidazole-5-carbonitrile system. The derivative was named LZ-106 (Figure 6) [69].

LZ-106 induced apoptosis via activation of the ROS-dependent DNA damage response in NSCLC (non-small-cell lung cancer) in both cultured cells and in a xenograft mouse model. The authors demonstrated that LZ-106 notably induced ROS overproduction and DDR. Interestingly, additional evidence in their findings revealed that DDR and apoptosis could be alleviated in the presence of an ROS scavenger, N-acetyl-cysteine (NAC), indicating ROS-dependent DDR involvement in the LZ-106-induced apoptosis. This research not only offered a new therapeutic candidate for NSCLC, but also gave new insights into the pharmacological research of quinolones [69].

In 2019, Yang et al. continued to investigate the mechanism of the cytotoxic activity of LZ-106. They substantiated the involvement of P53 activation in intracellular ROS generation upon LZ-106 treatment and related P53 to the ROS-induced viability inhibition and apoptosis, which was presented in previous research. P53 was shown to play an indispensable role in the upregulation of intracellular ROS in LZ-106-treated NSCLC cells through ROS detection. The authors further identified the antiproliferation effect of LZ-106 in NSCLC cells exhibited through G1 phase cell cycle arrest with a cell cycle analysis, with an expression analysis of the key proteins, and discovered that the cell cycle arrest effect is also mediated by the induction of ROS in a P53-dependent manner. In addition, the tumor-suppressive effect exhibited in vivo was demonstrated to be similar to that in vitro, which requires the participation of P53. Thus, LZ-106 is a potent antitumor drug possessing potent proliferation inhibition and apoptosis induction abilities through P53-dependent ROS modulation both in vitro and in vivo [101].

**Figure 7:**
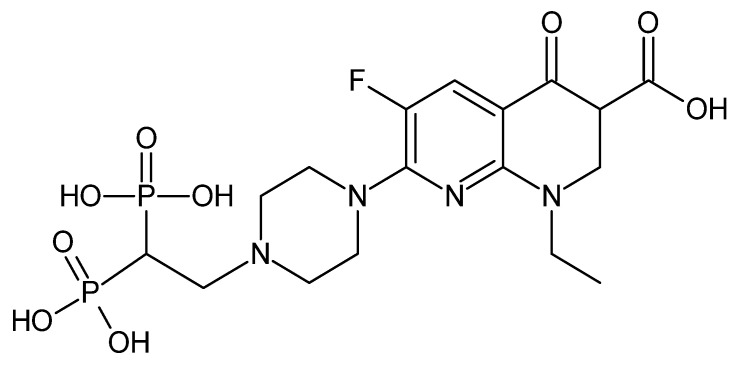
Structure of bis-enoxacin.

In 2018, Vracar et al. showed that enoxacin and bis-enoxacin (Figure 7) stimulate 4T1 murine breast cancer cells to release extracellular vesicles that inhibit osteoclastogenesis. 

Bis-enoxacin (BE) did not induce apoptosis or inhibit 4T1 murine breast cancer cell proliferation; however, it altered the release of extracellular vesicles and altered the miRNA expression. It also increased the number of GW/P bodies. Both enoxacin and bis-enoxacin at a concentration of 50 µM increased the number of GW/P bodies, but there were minimal changes in microRNA levels. Extracellular vesicles (EVs) released from 4T1 cells treated with 50 µM enoxacin or 50 µM bis-enoxacin stimulated the proliferation of RAW 264.7 cells, and both drugs significantly inhibited osteoclastogenesis in calcitriol-stimulated mouse marrow. EVs from 4T1 cells treated with enoxacin and bis-enoxacin displayed small reductions in the amounts of miRNA-146a-5p and let-7b-5p. In marked contrast, miRNA-214-3p, which has been shown to regulate bone remodeling, was increased 22-fold and 30-fold, respectively. We conclude that enoxacin and bis-enoxacin trigger in 4T1 cancer cells the release of EVs that inhibit osteoclastogenesis [49]. Similarly to the parent drug, bis-enoxacin induced the inhibition of osteoclast formation and bone resorption in primary mouse bone morrow cells via the inhibition of the binding between the B subunit of V-ATPase. Interestingly, the introduction of a bisphosphonate moiety increased the bone binding affinity [102]. Another confirmed mechanism of osteoclast formation was the interference of the JNK pathway in a rat model [103]. However, unlike ENX, its derivative increased the caspase 3 activity in osteoclast-like cells [82]. 

**Figure 8:**
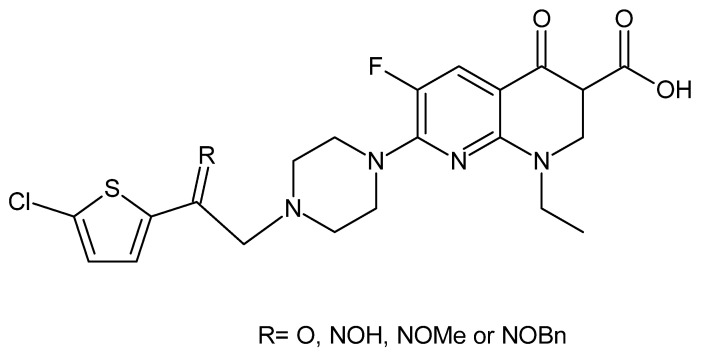
General structure of enoxacin derivatives prepared by Foroumadi et al. [88].

The introduction of 2-(5-chlorothiophen-2-yl)ethyl into the piperazine ring of enoxacin (Figure 8) increases its cytotoxicity against various cancer cell lines (melanoma, breast, epidermoid, colon, cervical and bladder carcinoma) compared to unmodified enoxacin. Foroumadi et al. obtained four new derivatives of enoxacin [88].

The synthetized derivatives differ from each other in the modification of the structure of an ethyl spacer. The introduction of oxygen into the ethyl spacer increased the cytotoxic activity from 137–196 µM for enoxacin to 137–131 µM for the oxygen derivative, which was a relatively minor change. The introduction of NOH or NOMe led to the strongest cytotoxic activity vs. all tested cell lines, with a IC_50_ range for NOH of ca. 3–10 µM and a IC_50_ range for NOMe of ca. 3–20 µM (depending on the cell line). A similar IC_50_ range of 2–14 µM for NOBn derivatives was observed in melanoma, epidermoid, cervical and bladder carcinoma cells. The IC_50_ values of NOBn derivatives in other cell lines and those of oxygen in all tested cell lines were much higher, but still significantly lower than the IC_50_ of unmodified enoxacin. To sum up, the introduction of the 2-(5-chlorothio-phen-2-yl)ethyl moiety into the 4′ position of enoxacin increased the cytotoxicity of the compound; however, the strength of the effect depended on the structure of the ethyl spacer. Introducing another nitrogen atom (R = NOH, NOMe or NOBe) led to a significant increase in antitumor activity [88]. 

**Figure 9:**
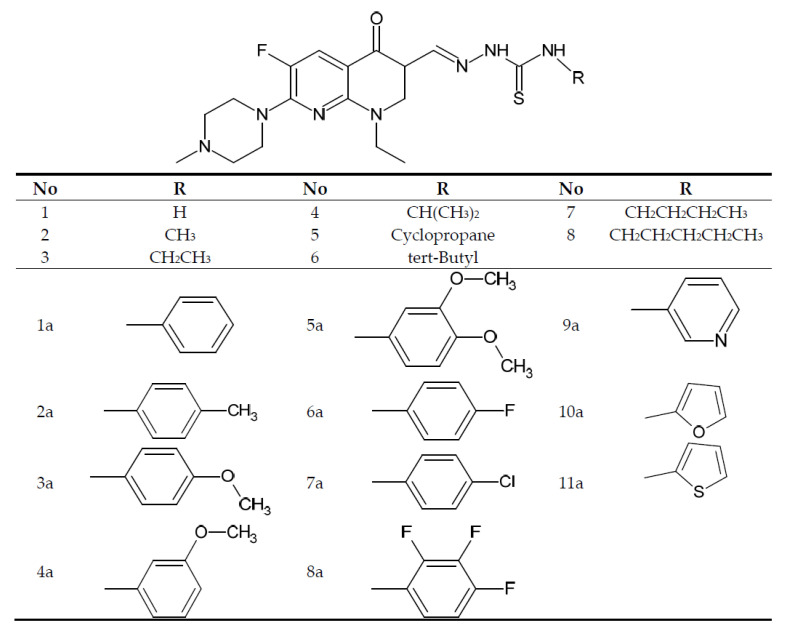
General structure of enoxacin alkyl (1–8) and aryl (1a–11a) derivatives prepared by Hu et al. [104] and Jiang et al. [105].

A series of enoxacin derivatives was formulated via the transformation of the carboxyl group in the skeleton of N-methylenoxacin (position 3) to an aldehyde group, followed by a direct reaction with N-arylhydrazinecarbothioamide (thiosemicarbazide) (CN106674220) or subjected to a condensation reaction with methyl hydrazinodithioformate and next to a nucleophilic substitution reaction with some N-alkylamines [104,105]. The structures of the patented 4-aryl thiosemicarbazone and 4-alkyl derivatives of enoxacin are presented below (Figure 9).

The obtained derivatives were tested for antitumor activity on cancer cell lines, including Hep-3B (human liver cell line containing integrated hepatis B virus genome), PANC-1 (human pancreatic cancer cell line) and HL-60 (human leukemia cell line). The cytotoxic activity against cancer cell lines was compared to that against a normal cell line—Vero (from the kidney of a normal adult African green monkey). 

The alkyl derivatives showed cytotoxicity within limits (IC_50_ 6.0–20.5 µM). The most active derivative was the derivative with the cyclopropyl substituent in the tiosemicarbazide part. Extending the alkyl substituent, especially above four carbon atoms, resulted in lower cytotoxic activity. All derivatives were characterized by significantly lower cytotoxicity against the normal Vero cell line (over 66.5 µM) [104].

The aryl derivatives, similar to the alkyl ones, were characterized by much higher cytotoxicity compared to enoxacin, in the range of 2.7 to 33.5 µM. Derivatives 1a–5a and 7a are characterized by more than two times lower cytotoxic activity compared to derivatives 6a and 8a–11a. The introduction of fluoro substituents to the phenyl ring or the replacement of the phenyl substituent with pyridyl, furanyl and thiophenyl substituents increases the cytotoxic activity. The most active compound was the derivative containing a pyridyl substituent in the tiosemicarbazide part (IC_50_ 3.2–6.2 µM). In this case, all derivatives were also characterized by significantly lower cytotoxicity compared to the normal Vero cell line (above 73.4 µM) [105].

**Figure 10:**
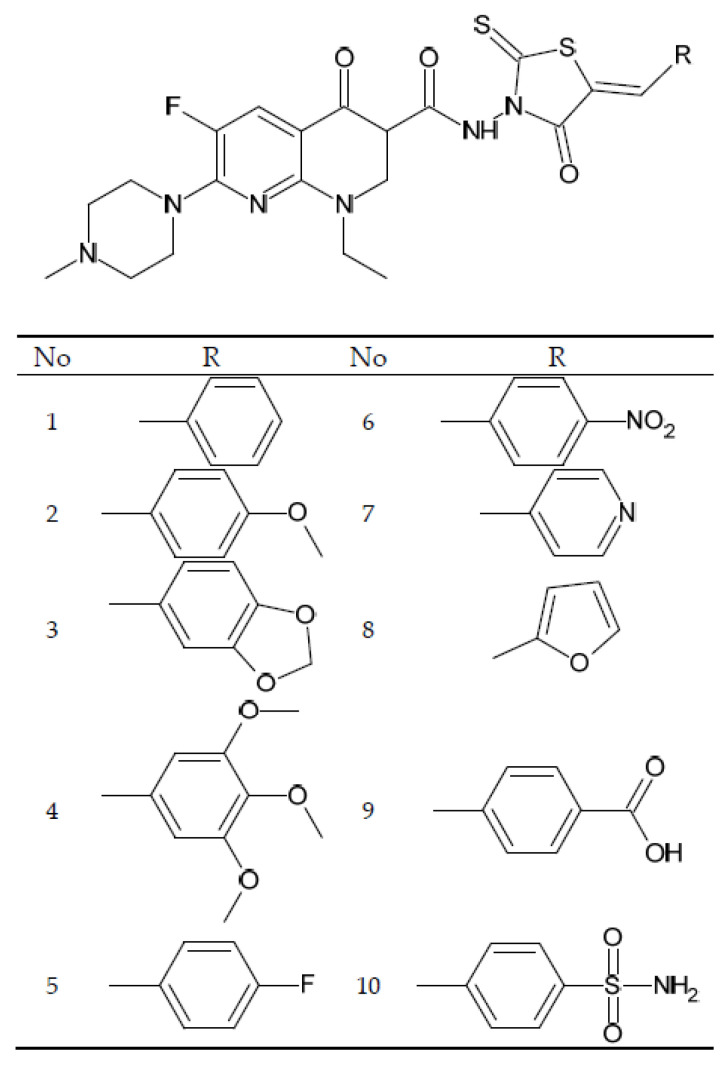
General structure of enoxacin alkyl derivatives prepared by Wang et al. [106].

Chinese scientists have tested the cytotoxic activity of some rhodanine derivatives of N-methylenoxacin (Figure 10). The methylated enoxacin in the reaction with hydrazine gave hydrazide, which next was transformed into rhodanine derivatives. The newly synthetized compounds were tested for antitumor activity on the cancer cell lines Hep-3B, PANC-1 and HL-60, and the Vero cell line was used as a normal cell line. The aryl derivatives with a 1,3-thiazolidene-2,4-dione ring, similar to those previously tested, were characterized by much higher cytotoxicity compared to enoxacin in the range of IC_50_ 1.8–21.5 µM. The derivatives numbered 1–4 are characterized by more than two times lower cytotoxic activity compared to the derivatives numbered 5–10. The most active derivative was the derivative containing a benzenesulfonamide substituent in the rhodanine part (10). In this case, all derivatives were also characterized by a significantly lower cytotoxicity compared to the normal Vero cell line (above 58.3 µM) [106].

**Figure 11:**
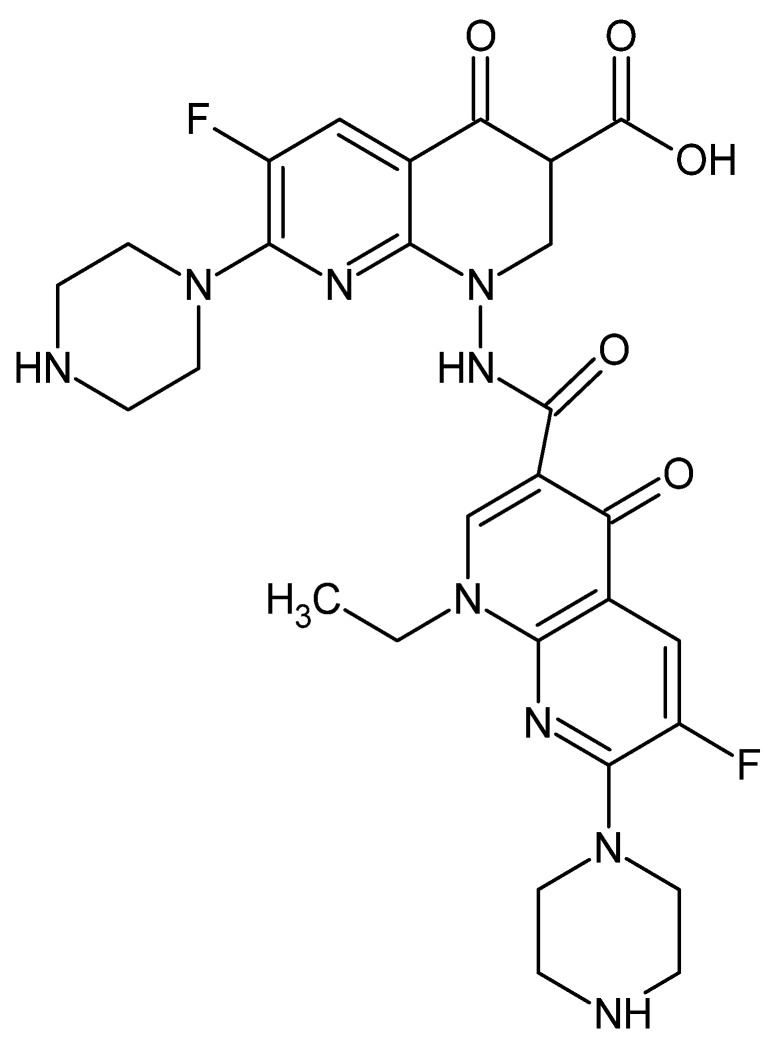
General structure of enoxacin alkyl derivatives prepared by Li et al. [107].

Another patented derivative was a dimeric form of enoxacin. The 1-(N-enoxacin amide)-enoxacin derivative (Figure 11) was obtained in three step sequence reactions: the condensation of enoxacin hydrazine with nalidixic acid derivative, the introduction of a piperazine ring and the final hydrolysis of a carboxylic group. The obtained compound was characterized by cytotoxicity within the limits (3.6–7.6 µM) against the neoplastic cell lines (Hep-3B, PANC-1, HL-60) and significantly lower cytotoxicity against the normal Vero cell line (72.8 µM) [107].

## 6. Conclusions

Enoxacin is a multifaceted drug. It is not only an effective antibiotic but also a promising anticancer agent. Unlike other fluoroquinolones, ENX exhibits extraordinary cytotoxic mechanisms of action. The presence of a carboxyl group at the third carbon atom, fluorine at the seventh carbon atom, as well as nitrogen at the eighth position of naphyridine is crucial for the regulation of miRNA abundance. ENX has the potential for the dysregulation of miRNA biogenesis by interfering with the PIWIL3 protein or DHX9 helicase, a part of the RNA-induced silencing complex (RISC), as well as the RISC-loading complex proteins DICER and TRBP. Additionally, it induced an elevated number of GW/P bodies. The alteration of those pathways was confirmed in the enoxacin-induced cytotoxicity towards colorectal, breast, prostate, thyroid and Ewing’s sarcoma family (ESFT) cancer cells. Changes in miRNA biogenesis resulted also in a decrease in inflammatory response in mice, affecting the neurobiology, including in neuronal differentiation and the inhibition of osteoclastogenesis. Another confirmed mechanism of osteoclast bone resorption inhibition was the suppression of the JNK signaling pathway and binding with V-ATPase subunits. Moreover, it has been described as a convenient tool for the regulation of CRISPR-Cas9 activity. Another anticancer mechanism was the induction of DNA damage by ROS stress generation. Enoxacin caused an induction of oxidative stress by singlet oxygen but not by superoxide radicals. Interestingly, it induced morphological changes, cell cycle arrest, growth inhibition, a decrease in invasiveness and apoptosis in cancer but not in normal cells. The above findings give evidence for the use of enoxacin, especially in bladder cancer therapy, where the achievable urine concentrations are far higher than IC50 and can be maintained constantly for a long time period. Furthermore, this type of cancer could be harnessed using two sources of enoxacin—urine and blood.

The modification of the piperazine ring or the carboxylic group resulted in enhanced cytotoxicity comparing to free enoxacin. Some of the derivatives were proven to cause cell cycle arrest and the induction of oxidative stress, while others dysregulated the levels of miRNA. Interestingly, the LZ-106 derivative caused the formation of superoxide radicals, in contrast to the parent drug. Unfortunately there are no clinical studies on enoxacin’s anticancer activity, while the results from in vitro studies are scarce compared to other fluoroquinolones. 

Taken together, enoxacin has anticancer potential, and its modifications may bring additional therapeutic benefits.

## Abbreviations

8-oxodGuo	8-Oxo-7,8-dihydro-2′-deoxyguanosine
ALA	δ-aminolevulinic acid
amiRNAs	artificial miRNAs
ATF6	activating transcription factor 6
Bax	Bcl-2-associated X protein
Bcl-2	B-cell lymphoma 2
BE	bis-enoxacin
CHOP	C/EBP homologous protein also knows as DNA damage-inducible transcript 3
CSC	cancer stem–like cells
DDR	DNA damage response
DICER	endoribonuclease DICER
dnTGFβRII	dominant negative TGF-β receptor
Drosha	type III RNase Drosha
EC50	effective concentration
EMT	epithelial–mesenchymal transition
ENX	enoxacin
ESFT	Ewing’s sarcoma family tumor
EVs	extracellular vesicles
FACS	fluorescence-activated cell sorting
GW/P-bodies	GW-Processing bodies
IRE1	inositol-requiring enzyme 1
MCL-1	induced myeloid leukemia cell differentiation protein
METTL3	methyltransferase-like 3
miRNA	microRNA
MMP2	matrix metalloproteinase-2
mRNA	messengerRNA
NAC	N-acetyl-cysteine
NOXA	phorbol-12-myristate-13-acetate-induced protein 1 also known as Noxa
NSCLC	non-small-celllungcancer
piRNA	Piwi-interacting RNA
PIWIL3	Piwi-like protein 3
PolII	polymerase II
pri-miRNA	primary transcript
Ran	GTP-binding nuclear protein Ran
RISC	RNA-induced silencing complex
ROS	reactive oxygen species
sgRNA	single-guide RNA
SMER	small-molecule enhancer of microRNA
SN-38	7-ethyl-10-hydroxy-camptothecin
TLR	Toll-like receptor
TRBP	TAR RNA-binding protein 2
V-ATPase	vacuolar H+-ATPase
**Cell Lines List**	
Human Cancer Cells	
breast cancer	MCF7
cervical cancer	HeLa, C33A
colorectal cancer	Co115, RKO and HCT-116
epidermoid carcinoma	A431
Ewing’s sarcoma family tumor (ESFT)	A673, TC252, STA-ET-8.2
leukemia	HL-60
liver cancer	Hep-3B
lymphoma	WTK-1
melanoma	A375, Mel-Juso, Mel-Ho
non-small cell lung	H460, A549, PC9
ovarian cancer	A2780
pancreatic cancer	AsPC1, PANC-1
prostate cancer	DU145, LNCaP, VCaP, PC-3, 22Rv1, Co115
thyroid cancer	Cal62, TPC1, SW1736
human non-cancer cells	
embryonic kidney	HEK 293
keratinocyte	HaCaT
primary pediatric mesenchymal stem cells	hpMSCs
animal cells	
murine macrophage	Raw 264.7
murine breast cancer cells	4T1
African green monkey’s normal kidney cells	Vero
